# Characterization of B-Genome Specific High Copy hAT MITE Families in *Brassica nigra* Genome

**DOI:** 10.3389/fpls.2020.01104

**Published:** 2020-07-21

**Authors:** Sampath Perumal, Brian James, Lily Tang, Sateesh Kagale, Stephen J. Robinson, Tae-Jin Yang, Isobel A. P. Parkin

**Affiliations:** ^1^ Agriculture and Agri-Food Canada, Saskatoon, SK, Canada; ^2^ National Research Council Canada, Saskatoon, SK, Canada; ^3^ Department of Plant Science, Plant Genomics and Breeding Institute, and Research Institute of Agriculture and Life Sciences, College of Agriculture and Life Sciences, Seoul National University, Seoul, South Korea

**Keywords:** *Brassica nigra* (black mustard), transposons (TE—transposable elements), hAT family, *Brassica*, miniature inverted-repeat transposable elements (MITEs)

## Abstract

Miniature inverted-repeat transposable elements (MITEs) are non-autonomous class II transposons which have been shown to influence genome evolution. *Brassica nigra* L. (B-genome) is one of three *Brassica* diploids cultivated primarily as an oil crop, which harbors novel alleles important for breeding. Two new high copy hAT MITE families (BniHAT-1 and BniHAT-2) from the B-genome were characterized and their prevalence assessed in the genomes of the related diploids, *rapa* L. (A) and *Brassica oleracea* L. (C). Both novel MITE families were present at high copy numbers in the B-genome with 434 and 331 copies of BniHAT-1 and BniHAT-2, respectively. Yet less than 20 elements were identified in the genome assemblies of the A, and C -genomes, supporting B-genome specific proliferation of these MITE families. Although apparently randomly distributed across the genome, 68 and 70% of the B-genome MITEs were present within 2 kb flanking regions of annotated genes suggesting they might influence gene expression and/or function. In addition, MITE derived microRNAs and transcription factor binding sites suggested a putative role in gene regulation. Age of insertion analysis revealed that the major proliferation of these elements occurred during 2–3 million years ago. Additionally, site-specific polymorphism analyses showed that 44% MITEs were undergoing active amplification into the B-genome. Overall, this study provides a comprehensive analysis of two high copy MITE families, which were specifically amplified in the B-genome, suggesting a potential role in shaping the *Brassica* B-genome.

## Introduction

Transposable elements (TEs) constitute a major fraction of most eukaryotic genomes; for instance more than 85 and 71% of the *Triticum aestivum* and *Aedes albopictus* genome, respectively were occupied by TEs (Lee and Kim, 2014; Chen et al., 2015; Appels et al., 2018). Based on the mechanism of transposition TEs are typically classified into class I TEs (Retro-transposons) and class II TEs (DNA transposons). Class I TEs are mobilized into a new position of the same genome by a copy-and-paste mechanism through an RNA-intermediate, while class II TEs are mobilized through a cut-and-paste mechanism. Autonomous TEs have functional coding regions allowing independent transposition while those lacking this ability are non-autonomous. Transposition of TEs catalyzed by transposases into different genomic regions can have a significant impact on gene structure, expression and function and ultimately may influence genome adaptation and evolution (Wicker et al., 2007; Sampath et al., 2015; Vicient and Casacuberta, 2017).

Miniature inverted-repeat transposable elements (MITEs) are non-autonomous class II DNA transposons, usually small (< 1000 bp) in size, AT-rich, and ubiquitously present in almost all plant genomes (Pritham, 2009; Bennetzen and Wang, 2014; Sampath and Yang, 2014). Each MITE contains signature structures known as terminal inverted repeats (TIRs ≥10 bp) at either end flanked by target site duplications (TSDs, 2–10 bp) (Fattash et al., 2013). MITEs are deletion derivatives derived from autonomous TEs thus share structure and sequence similarity with their parent element; for example a *Tourist* superfamily MITE, *mPing*, is derived from *ping* DNA transposons (Feschotte et al., 2002; Naito et al., 2009). Conversely, some MITE families, such as the *stowaway* MITE superfamily may have originated through cross mobilization facilitated by distantly related TEs such as *Marinar* like elements (Feschotte et al., 2005; Macko-Podgórni et al., 2019). Regardless of their size and origin and their requirement for trans-acting transposases, MITEs tend to be present in high copy numbers. In rice MITEs make up 10% of the total genome, consisting of 179,415 elements from 339 families (Chen et al., 2013). Though studies have suggested that MITEs are formed through usurping the endogenous gap repair mechanism, it is still unclear how MITE copy numbers increase (Naito et al., 2009).

MITEs are classified into 15 different superfamilies based on their TSDs in plant and animal genomes. So far seven superfamilies of MITE, Tcl/mariner, PIF/Harbinger, hAT, Mutator, CACTA, P-element, and Novosib, have been found in plants whereas other superfamilies were common in animals (Wicker et al., 2007; Chen et al., 2013). The hAT family has been investigated in many plant species including *Zea maya*, *Orzya sativa*, *Arabidopsis thaliana*, and *Brassica* species (Bundock and Hooykaas, 2005; Muehlbauer et al., 2006; Benjak et al., 2008; Menzel et al., 2012; Chen et al., 2013; Menzel et al., 2014; Sampath et al., 2014; Nouroz et al., 2015b) and is among the most prevalent of such elements, of those *Brassicaceae* species studied between 0.7 and 4.5% of the total genome length were covered by MITE species (Chen et al., 2013). Maize kernel color changing factor *Activator* (Ac), an autonomous hAT transposon was the first TE discovered followed by its non-autonomous partner element *Dissociation* (Ds) (Feschotte et al., 2002). Members of the hAT superfamily have been found in various distantly related organisms, suggesting their ancient origin, which predates the divergence of plant-fungi and animals (Kempken and Windhofer, 2001; Rubin et al., 2001). The extensive P-MITE database provides a collection of MITE sequences from 41 plant species that includes 3,527 families from 7 superfamilies (Chen et al., 2013). MITEs have been shown to be distributed into almost all genomic regions, although some MITE families have a tendency to closely associate with genes (Guo et al., 2017). Insertion of MITEs into various genic and near genic-regions can impact regulation of genes and genome evolution (Oki et al., 2008; Naito et al., 2009). Various studies have suggested that MITEs play a direct role in transcriptional and post-transcriptional gene modifications by acting as an exon, a source of small RNAs, or providing the transcription start site and the poly(A)-tail (Naito et al., 2009; Sampath et al., 2013). Furthermore, their high copy and stable inheritance make MITEs a valuable tool for marker development (Monden et al., 2009; Sampath et al., 2015).

The genus *Brassica* (family *Brassicaceae*) is an economically important source of vegetable, oilseed and fodder crops (Cheng et al., 2017). The evolutionary relationship of the six *Brassica* species including the three diploid species, *Brassica rapa* L. (A-genome, 2n=2×=485 Mb), *B. nigra* L. (B, 2n=2×=600 Mb) and *B. oleracea* L. (C, 2n=2×=630 Mb) and derived allotetraploids *B. juncea* (L.) Czern. (AB, 2n=4×=1100 Mb), *B. carinata* A. Braun (BC, 2n=2×=1230 Mb) and *B. napus* L. (AC, 2n=2×=1120 Mb) was depicted by the triangle of U (Nagaharu, 1935). The recent availability of whole genome sequences for all species (except BC) has provided an unprecedented opportunity to study elements of genome structure and carry out comparative analysis (Wang et al., 2011; Chalhoub et al., 2014; Liu et al., 2014; Parkin et al., 2014; Yang et al., 2016). Though the B-genome has comparatively less economic importance than the A and C genomes, it comprises a pool of novel alleles conferring numerous elite characteristics for traits such as diseases resistance, salt and drought tolerance, which can be used for trait improvement in the valuable oilseed *B. napus* (Truco and Quiros, 1994). Genome sequencing of the A and C-genomes revealed that about 40–60% of the genome was occupied by repeat sequences including TEs and tandem repeats (Wang et al., 2011; Chalhoub et al., 2014; Liu et al., 2014; Parkin et al., 2014; Yang et al., 2016). While there have been a few studies of MITEs in *Brassica* genomes, there has as yet been no equivalent analysis of the B-genome (Nouroz et al., 2015a). In the current study, through comparison of 170 candidate MITE families between the diploid genomes two hAT MITE families which proliferated specifically in the B-genome were identified. Here, we characterized the two hAT MITE families and their distribution and potential evolutionary impact on the *Brassica* B-genome is discussed.

## Materials and Methods

### Identification of MITE Families From *B. nigra* Genome

A newly developed *B. nigra* whole genome pseudo-chromosome assembly (Ni100-LR) derived from Nanopore read data was used, which with unanchored scaffolds covered 503.5 mega bases (Mb) (Perumal et al., 2020)^1^. MITE Digger was used with default parameters (Yang, 2013) and identified 234 candidate MITE families. In addition, MITE finderII (Hu et al., 2018) was applied with default parameters, which identified 224 potential families, of these 170 candidate MITEs were annotated with both programs and used for further analyses. MITE signature structures such as TIRs and TSDs were characterized using the selfBLAST tool from NCBI^2^. Candidate MITE families were searched against Repbase and P-MITE database (Chen et al., 2013; Bao et al., 2015) to identify homologous MITEs in other plant genomes. MITE-derived microRNAs, were identified by searching MITE sequences from the two families against the available microRNA database, miRbase (version19)^3^ with default parameters for embryophyta genomes (Kozomara and Griffiths-Jones, 2013). Secondary structure of MITEs was created using the Mfold software program (Zuker, 2003). Putative transcription factor binding sites (TFBS) were identified from the MITE sequences using PROMO^4^ for genomes of embryophyta (Messeguer et al., 2002).

### Distribution and Phylogenetic Analysis of MITE Members in A, B, and C-Genomes

In addition to the B-genome, a whole genome assembly for *B. rapa* (389.2 Mb) *V 1.5*, *B. oleracea* (488 Mb) Version 1.0 and *Arabidopsis thaliana* TAIR 10 (125 MB) were obtained from BRAD (Cheng et al., 2011), Ensembl (https://plants.ensembl.org/Brassica_oleracea/Info/Index) and TAIR (Huala et al., 2001), respectively. Furthermore to assess genome specificity, MITE members were extracted from available genome sequences of the *Brassica* allotetraploids, *B. juncea* (Yang et al., 2016) and *B. napus* (Chalhoub et al., 2014). Related MITEs were identified from the reference genomes based on two hAT families using BLASTn (E-value of E^-05^), those with ≥ 80% sequence alignment length and identity were considered intact MITEs and extracted from their respective genome. The position of MITE insertion on the B -genome relative to gene annotation was compared using a combination of bedtools and shell scripts. Intact MITEs were used for phylogenetic analysis. ClustalW alignment of MITE members of each family and phylogenetic trees were generated using the neighbor-joining method with 1,000 bootstrap replications in MEGA X (Kumar et al., 2018).

### MITE Copy Numbers in the *Brassica* A, B, and C Genomes

MITE copy numbers were estimated in the three *Brassica* genomes using the previously described read depth approach (Waminal et al., 2015). Paired-reads from 11 *Brassica* accessions including *B. rapa, B. nigra*, and *B. oleracea* were obtained, accessions and data sources are detailed in **Table S1** (Chalhoub et al., 2014; Waminal et al., 2015). Using the CLC reference map tool included in CLC Assembly Cell (5.0.2.), whole genome shot-gun (WGS) reads were mapped against the MITE sequences to quantify the abundance in a haploid genome with the threshold level of more than 80% identity across more than 50% of the read length. Overall read depth was normalized to haploid genome coverage for all three diploid *Brassica* genomes based on corresponding genome sizes.

### Estimating MITE Insertion Time

The divergence rate between the individual members and their consensus sequences can be used to estimate the age of the element (Jiang et al., 2016). In order to estimate the age of the two MITE families, multiple sequence alignment of members and consensus sequences for each MITE family was carried out using clustalw. In order to avoid bias towards the more numerous subfamilies equal numbers of elements were used from each subfamily/clade to create the consensus. For example, for BniHAT-1 the consensus was created with 75 random members from BniHAT-1 clade I along with 75 members of BniHAT-1 clade II. Likewise, 69 members from clade II with all the members from clade I, III, and IV were used to create a consensus for BniHAT-2. Kimura 2-parameter distance method implemented in the MEGA X program was used to estimate the level of base substitution rate per site (*k*) between each MITE element and the consensus sequence (Kimura, 1980). Finally, MITE insertion time was then estimated using the formula *T* = *k*/2*r*, assuming *r* = 1.30 × 10^−8^ (Ma and Jackson, 2006).

### Analysis of MITE Insertion Polymorphism (MIP)

Site-specific polymorphism or MITE insertion polymorphism (MIP) was analyzed for 22 different *Brassica* accessions to identify the presence (inserted site) or absence (empty site) and activity of a MITE in a specific genomic location (Sampath and Yang, 2014). Total DNA from the 22 accessions was extracted from fresh leaves based on the modified CTAB method (Allen et al., 2006). Accessions used for the MIP analysis included four *B. rapa* (A1-A4), fourteen *B. nigra* (B1-B14) and four *B. oleracea* (C1-C4) as described in **Table S2**. MITE flanking primers were designed using Primer3 for 60 target regions distributed over the B-genome (Rozen and Skaletsky, 2000). Primer sequences and their expected product size and gel profile information are listed in **Table S3**. PCR was performed in a 10 µl total reaction volume consisting of 5 ng DNA concentration, 0.2 µM of each primer, 1 × PCR buffer, 2.5 µM dNTPs, and 1 unit *Taq* DNA polymerase (Invitrogen, CA). PCR was carried out with the following conditions; 5 min at 94°C, 35 cycles of 95°C for 1 min, 57°C for 30 s, and 72°C for 1 min, with a final extension at 72°C for 5 mins. PCR products were separated by electrophoresis in 2% agarose gels with 1 x TBE buffer, gels were pre-stained with GelRed and amplification products were visualised on a UV trans-illuminator.

## Results

### Characterization of Two High Copy hAT Families in the B-Genome

The recently developed B-genome pseudo-chromosome assembly (Ni100-LR) was used for the characterization of MITEs (Perumal et al., 2020). Mining of MITE families using MITE Digger and MITE FinderII identified 170 candidate MITE families accounting for approximately 1.2% (6.3 Mb) of the B-genome (**Table S4**). Comparative analysis of the relative copy number of the 170 MITE families from the three *Brassica* diploid genomes (A, B, C-genomes) revealed two MITE elements with high copy numbers in the B-genome compared to the A and C-genomes (**Table 1**). Both elements were comparatively short in size (673 and 666 bp) with 25 and 12 bp TIRs, respectively (**Figure S1**). Following previous classifications, based on the characteristic 8 bp TSD, the elements were identified as part of the hAT superfamily (Wicker et al., 2007). Named BniHAT-1 and BniHAT-2, both elements had high AT-content, 70 and 75% respectively, which is typical of a MITE family. Furthermore, homology searches against related MITE elements in Repbase and the P-MITE database revealed BniHAT-1 had homology with hAT elements from the grapevine genome while BniHAT-2 had homology with elements from the *A. thaliana* (**Table 1**).

**Table 1:** Characteristics of the two *Brassica nigra* (Bni) hAT families.

MITE family	size (bp)	TIR (bp)	TSD (bp)	AT (%)	Intact copies in reference genomes						Homologous element (HE)	HE inP-MITE DB
					*B. rapa* (A)	*B. nigra* (B)	*B. oleracea* (C)	*B. juncea* (AB)	*B. napus* (AC)	*A. thaliana*		
BniHAT-1	673	25	8	70	1	434	1	432	1	1	VIHAT2-N1_VV	DTA_Brr74
BniHAT-2	666	12	8	75	3	331	18	200	1	5	ATHATN1	DTA_Brr81

Transposable element derived microRNAs have been shown to be involved in regulation of gene function by affecting destabilization and expression of mRNA. A search for MITE-derived microRNAs revealed a total of 11 different microRNAs, using an E-value of 1E^-10^, with six derived from the BniHAT-1 and five from the BniHAT-2 family (**Table S5**). The MITE-derived microRNAs were distributed randomly across the MITE sequences and five anti-sense microRNAs were also observed. Furthermore, predicted secondary structures for representative BniHAT MITE sequences suggested a mechanism for generation of the miRNAs (**Figure S2**). MITEs have been shown to influence transcriptional regulatory networks by providing novel transcription factor binding sites (TFBS) (Morata et al., 2018). Both MITE elements were found to contain 18 different potential TFBS that were enriched with stress responsive TFBS such as those for bZIP, MADS, and SBF1 transcription factors (**Table S6**). Studying the overall genome distribution of the 18 TFBS motifs revealed that the majority were found in TE space at levels which might be expected based on the repeat content of the genome; however, some appeared to be more prevalent in TE space, for example >78% of both the PHR1(Phosphate starvation response) and LIM1 (Cysteine rich zinc-binding) motifs were located in TE space. For the BniHAT elements, which occupy less than 0.001% of the genome, 24 and 16% of the LIM1 and AP3:PI (MADS box transcription factor) motifs, respectively were derived from the two BniHAT MITE families. This finding is in keeping with previous analyses suggesting a role for TE in controlling gene expression, further functional analysis would be required to confirm a specific role for the BniHAT elements (Kuang et al., 2009; Cui et al., 2017).

### Copy Number Analysis Based on Whole Genome Assembly and WGS Reads

Both MITE families were used to search the three diploid *Brassica* (A, B, C) and the *A.thaliana* (At) whole genome assemblies. BLASTn analysis of BniHAT-1 revealed 434 intact members in the B-genome, while only one element was found in each of the three other genomes. Likewise, for BniHAT-2, 331, 3, 18, and 5 elements were found in the B, A, C and At-genomes, respectively (**Table 1**). Compared to BniHAT-1, BniHAT-2 had slightly higher numbers in all the related genomes and was found to have its highest copy number in the *B. oleracea* genome (**Table 1**). Both of the MITE families showed B-genome specific proliferation with 434 to 18-fold difference. In addition, analysis of MITE members in the available *Brassica* B-genome containing allotetraploid *B. juncea* (AB) identified 432 and 200 members from BniHAT-1 and BniHAT-2, respectively. Of these, 533 in total were positioned on chromosomes, with 78 and 83% of BniHAT-1 and BniHAT-2 elements, respectively being present in the B-subgenome of *B. juncea*. The remaining chromosome anchored elements (79 BniHAT-1 and 29 BniHAT-2) were from the A-subgenome suggesting recent mobilization of these elements. In comparison, only 2 copies of BniHAT elements were found in the *B. napus* (AC) genome suggesting no amplification.

**Figure 1:**
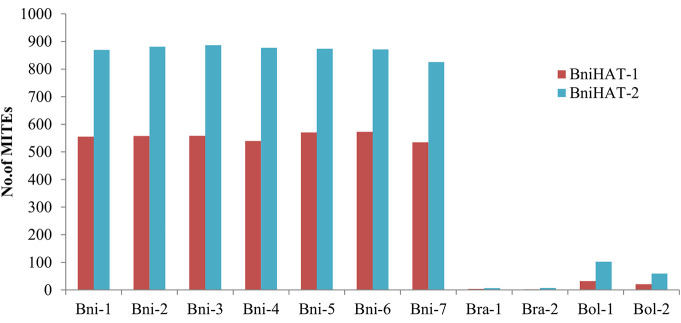
Estimation of MITE copy numbers in four diploid *Brassica* genomes (*B. nigra*: Bni, *B. rapa*: Bra, *B. oleracea*: Bol) based on read mapping using whole genome sequence reads.

Copy numbers were also estimated based on an WGS read depth approach for the *Brassica* diploid genomes. This revealed a similar pattern with that estimated using the whole genome assemblies, with 550, 10 and 25 BniHAT-1 and 850, 8 and 75 BniHAT-2 members in the B, A, and C-genome, respectively (**Figure 1**). While the B-genome has the highest copy numbers, with up to 20-fold differences, for both elements higher numbers were observed in the *Brassica* C-genome compared to the A (**Figure 1**).

### Genomic Distribution of MITEs

**Figure 2:**
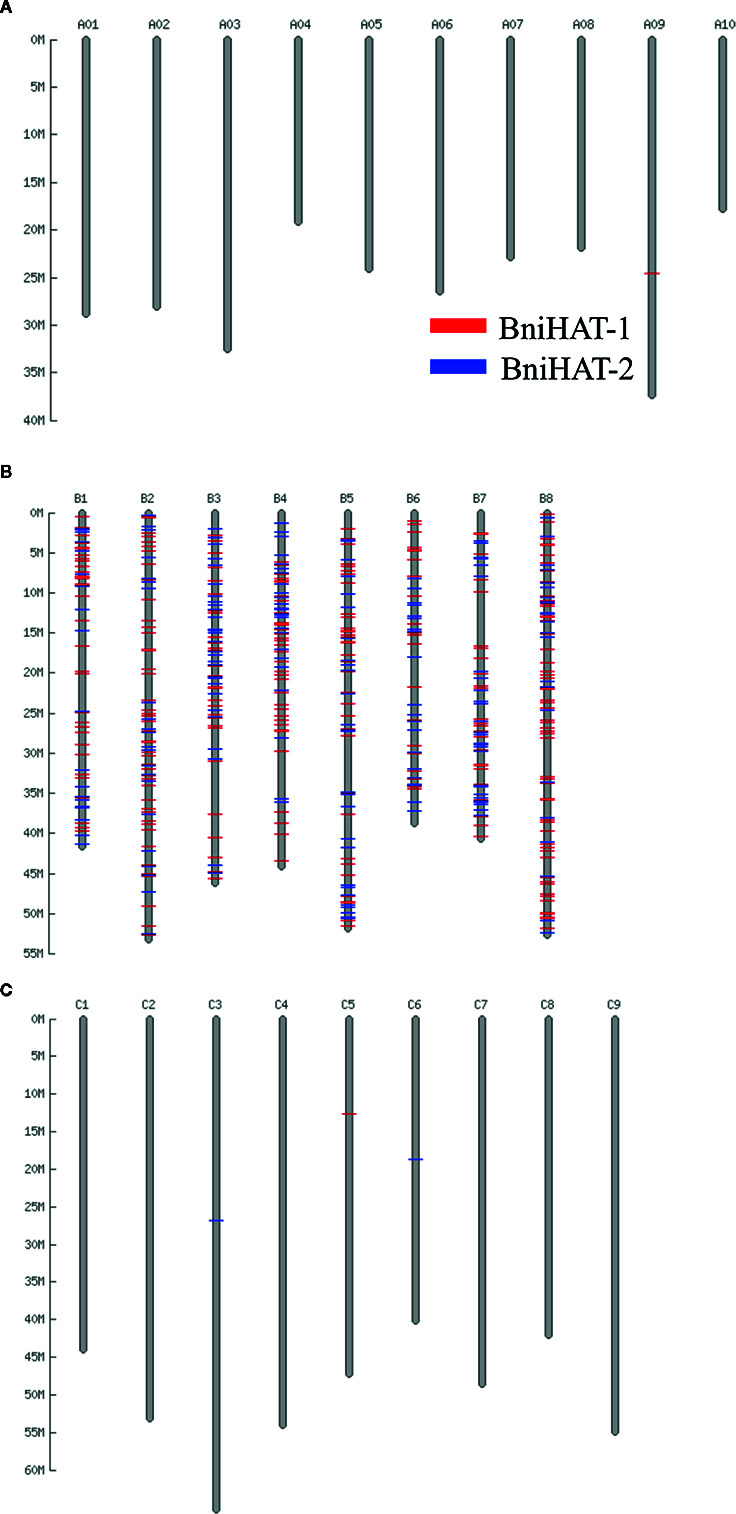
Distribution of two HAT family members across the pseudo-chromosomes of the *B. rapa* **(A)**, *B. nigra* **(B)** and *B. oleracea* **(C)** genome.

**Figure 3:**
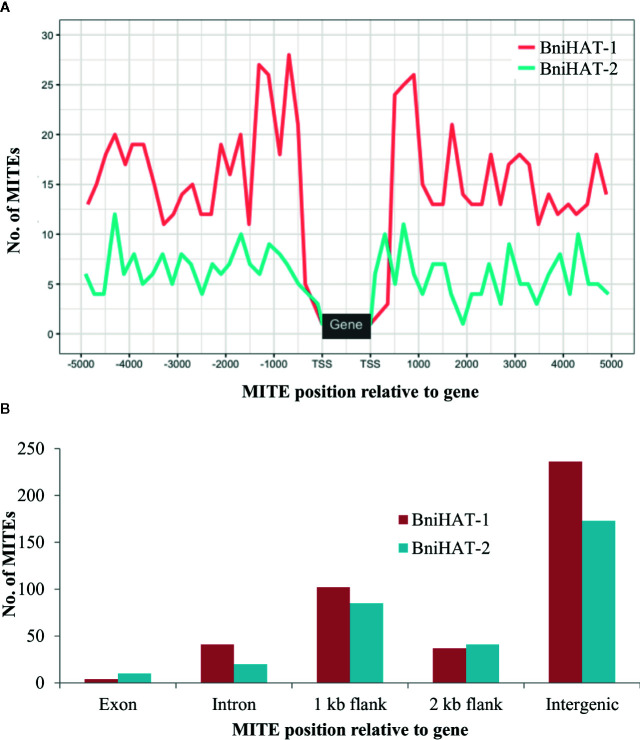
Genomic position of BniHAT-1 and BniHAT-2 elements in the *B. nigra* genome. **(A)** Plot showing distribution of MITEs within 5 Kb of the Transcription start/stop site. **(B)** Graph showing number of MITEs in each genomic position.

Both MITEs families appeared to show a random distribution across the B-genome chromosomes (**Figure 2**). The MITE insertion positions were characterized in the B-genome to check for any preferential association with particular genomic regions or features. Out of 434 and 331 members, 184 (44%) BniHAT-1 and 156 (47%) BniHAT-2, respectively were in close proximity to genes (≤ 2 kb flanking) (**Figure 3**; **Table S6**; **Table S7**). This suggested the preferential association of both MITE families with euchromatic regions, although only one and three members from the BniHAT-1 and BniHAT-2 MITE families, respectively were inserted into gene exons (**Figure 3**; **Table 2**).

**Table 2:** MITE Members from BniHAT-1 and BniHAT-2 inserted into exonic regions of the *B. nigra* genome.

ID	Chr#	Start	End	Size	E-value	*B. nigra* _gene	Orthologue (*A. thaliana*)	Function
BniHAT2-4	B1	53,961,873	53,962,535	667	0	BniB01g054500.2N.1	AT3G10520	Class 2 non-symbiotic hemoglobin
BniHAT2-233	B4	6,296,857	6,297,488	649	0	BniB04g013020.2N.1	AT5G43600	Allantoate Amidohydrolase 2
BniHAT2-259	B5	16,032,215	16,031,555	667	0	BniB05g032100.2N.1		
BniHAT1-375	B4	8,429,613	8,428,948	675	0	BniB04g016760.2N.1	AT5G44900	Toll-Interleukin-Resistance (TIR) domain family protein

### Phylogenetic Analysis and Age of the MITE Insertion

**Figure 4:**
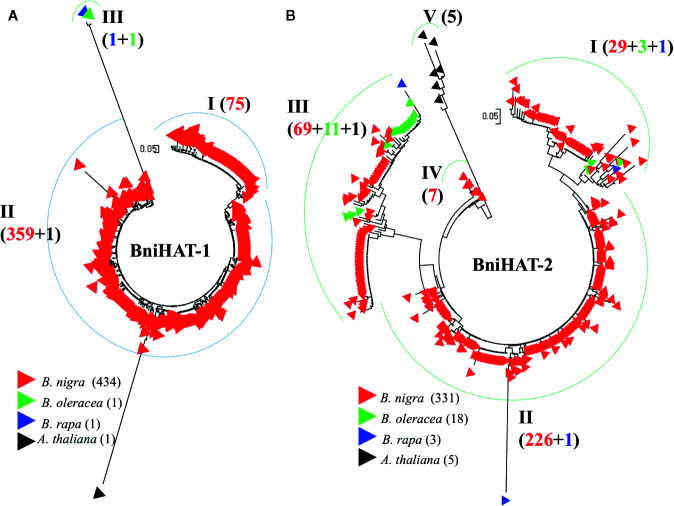
Phylogenetic analysis of BniHAT-1 **(A)** and BniHAT-2 **(B)** family members from the three diploid *Brassica* genomes and *A. thaliana*. The origin (color coded) and number of the different members from each of the four genomes is shown in parenthesis for each clade.

Phylogenetic analysis based on intact members from both MITE families reveals inter- and intra-genomic diversity for *Brassica* and the related species *A. thaliana*. BniHAT-1 family members showed a lower level of intra-species divergence compared to BniHAT-2 and a distant relationship with the small number of inter-specific elements (**Figure 4**). Three clades (I–III), including one clade containing the solitary A and C-genome members, can be observed from the phylogenetic analysis of the 437 BniHAT-1 family members. Clade I and II consist of 75 and 359 B-genome specific members, respectively, suggesting that members were amplified in a B-genome specific manner (**Figure 4A**). Likewise, phylogenetic analysis of 357 BniHAT-2 family members revealed five different clades (I–V) with 33, 227, 81, 7, and 5 members for each clade, respectively. BniHAT-2 members from *A. thaliana* were grouped into a separate clade from the *Brassica* genomes. Members from Clades I and III contained related C-genome elements, while Clade II consisted of 229 members from the B-genome, and a single member from the A-genome (**Figure 4B**).

**Figure 5:**
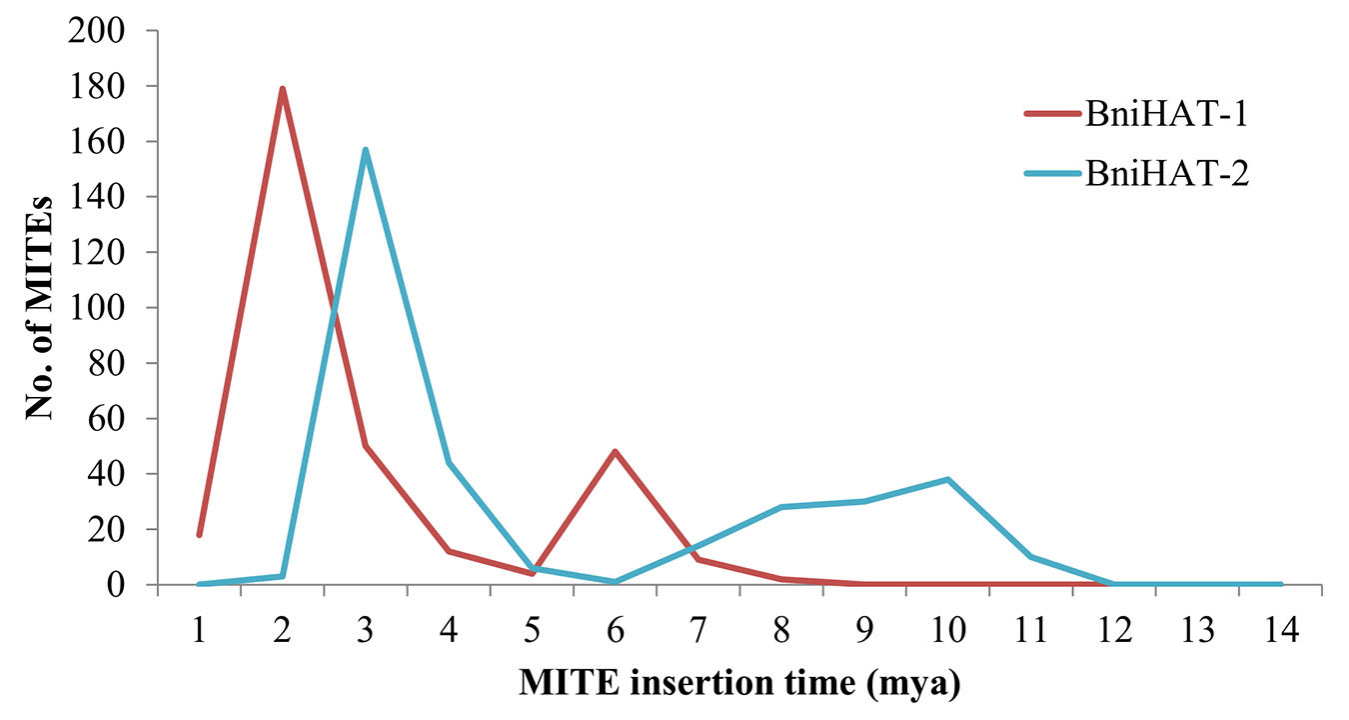
Age distribution of BniHAT-1 and BniHAT-2 family members in *B. nigra* genome.

The age(s) of the MITE elements were estimated to suggest the time of differential diversification. This revealed that the BniHAT-1 family has two bursts of amplification, a larger expansion about 2 million years ago (mya) and a smaller expansion about 6 mya. While BniHAT-2 family members showed a major proliferation of 150 members at approximately 3 mya with a less well defined event about 10 mya (**Figure 5**).

### Insertion Polymorphism of hAT Members in the Three Major Diploid *Brassica* Genomes

**Figure 6:**
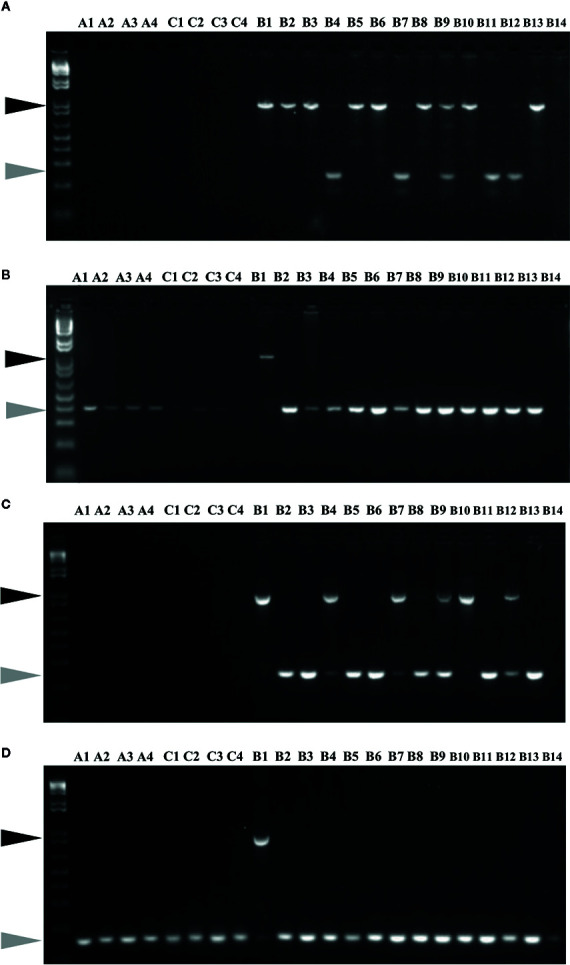
MITE insertion polymorphisms analyses of members from BniHAT-1 **(A, B)** and BniHAT-2 **(C, D)** families in three diploid *Brassica* genomes.

Insertion and potential activity of MITEs was studied using MITE insertion polymorphism (MIP) analysis, focusing on 60 specific sites in 22 *Brassica* accessions (**Figure 6**). Out of 60 targets analysed, which included 30 each from the two BniHAT families; 30 (100%) and 23 (77%) sites showed expected amplification, for BniHAT-1 and BniHAT-2 members, respectively. Overall, 52 out of the 53 amplified sites were specific to the B-genome and only one BniHAT-2 insertion was found in the C-genome, with no amplification found in the A-genome. MIP analysis revealed that 49 (92%) members appeared to be polymorphic in at least one accession. In addition, 13 out of 53 (25%) members showed evidence of recent insertions in the B-genome for two or more accessions (**Table S7**).

## Discussion

MITEs play an important role in gene and genome evolution by influencing gene structure and expression (Sampath and Yang, 2014). Taking advantage of the recently sequenced *B. nigra* B-genome, genome-wide characterization of MITEs was completed using the *denovo* MITE identification tools, MITE Digger and MITE finderII (Yang, 2013; Hu et al., 2018). Comparative analysis of the candidate elements revealed two MITE superfamilies of hAT transposons, which showed unique amplification in the *Brassica* B-genome compared to A and C-genomes. There have been various studies focusing on MITEs in *Brassica* genomes suggesting their evolutionary importance and also utility as source of markers (Chen et al., 2013; Sampath et al., 2013; Sampath et al., 2014; Nouroz et al., 2015a; Nouroz et al., 2015b). Though there is an extensive collection of MITEs for many plant genomes, including *B. rapa* and *B. oleracea*, very few elements have been subjected to in-depth structural and functional characterization (Chen et al., 2013). In addition, few studies on comparative analysis have included the B-genome (Nouroz et al., 2015a). This study provides the first in depth characterization of two largely B-genome specific MITE families.

MITEs are generally present in large quantities (hundreds of thousands of copies) per genome. An analysis of MITEs in 19 *Arabidopsis* accessions revealed 343,485 MITE-related sequences which contribute to a significant proportion of the genome, and impact the evolution of the genome (Guo et al., 2017). Similarly, genome-wide characterization of MITEs in *B. rapa* revealed 45,821 MITE-related sequences belonging to 174 families that are believed to influence genome structure and evolution (Chen et al., 2013). Furthermore, extensive characterization of MITEs in *B. rapa* revealed many relatively intact copies in the genome, for instance, the BraSto family was present in >1,500 intact copies per haploid genome (Sampath et al., 2013). Likewise, hAT superfamilies of MITEs were identified and characterized in various species including *B. rapa* and *B. oleracea*, *Oryza species*, *Musa species*, and *Beta vulgaris* and were found to be present at high copy numbers (Bundock and Hooykaas, 2005; Muehlbauer et al., 2006; Nouroz et al., 2015b). MITEs comprised approximately 1% of the *B. nigra* genome (Perumal et al., 2020), and in our analysis we identified two hAT families that are largely specific to the B-genome. Genome or lineage specific amplification of transposons including MITEs has been observed for many species (Feschotte et al., 2002; Choi et al., 2014) and has been suggested to play a role not only in increasing genome size but more specifically in genome adaptation (Parisod et al., 2010; Belyayev, 2014). Recent analysis of MITEs in multiple carrot genomes revealed extensive diversity in MITE insertion site polymorphism and differential association of particular MITE families with transcription factors, suggesting a role in gene regulation (Macko-Podgórni et al., 2019).

After polyploidization events in plants, bursts of transposon amplification have been found and thought to mitigate the effects of genome shock and gene dosage (Vicient and Casacuberta, 2017). In particular, bursts of transposition into various genic regions can take control of nearby gene expression for adaptation and genome evolution (Naito et al., 2009; Tenaillon et al., 2010). Furthermore, transposition bursts also influence structural changes of genes and genomes by subsequent inter-element recombination and chromosomal rearrangement, which can result in a decrease of genome size and loss of chromosomes as a long-term path to diploidization (Vicient and Casacuberta, 2017). This evolutionary response is unique for each transposable element family and each genome (Han et al., 2010; Lu et al., 2011). For example, characterization of TE types in *Gossypium* species revealed that different TE families with lineage-specific amplification caused variation in genome size (Hawkins et al., 2006). In *Brassica*, the centromeric associated PCRBr gypsy transposon specifically amplified in the A-genome (Lim et al., 2007). On the other hand, the B-genome does not have centromeric tandem repeats, which are common to A and C-genomes, suggesting a divergent evolutionary path (Lim et al., 2007; Koo et al., 2011). In this study, two MITEs were identified that specifically proliferated in the B-genome while few copies were found in the close relatives, implying the importance and potential influence of these MITEs on B-genome evolution. We also observed that BniHAT members are present at a low copy number in the A-subgenome of *B. juncea* suggesting active mobilization of BniHAT elements and implying a possible role in divergence of the allotetraploid sub-genomes.

MITEs can be activated by stress causing them to transpose into a different genomic location, while also amplifying their copy number; possibly by an abortive gap repair mechanism or by an unknown mechanism (Naito et al., 2009). Analysis of MITE age based on synonymous substitution rate revealed that both B-genome MITE families have a long and continuous evolutionary trajectory from 1–14 mya. Though both MITE families showed irregular and gradual amplification until 2 mya, the largest events occurred about 2–3 mya for both families. speculating a specific role of BniHAT families in B-genome evolution. The *Brassica* B-genome diverged 9 mya from the common ancestor of *B. rapa-oleracea*; independent amplification of the BniHAT elements in the B-genome suggest a role in genome adaptation and their close association with genic regions implicate their potential for impacting gene regulation.

MITEs have a tendency to distribute randomly across the genome, yet associate with genes or near genic regions and the distribution of MITEs into various genomic locations such as exon, intron and regulatory regions has the ability to influence gene structure, function and evolution (Naito et al., 2009). Based on our analysis, a significant proportion of members from both B-genome families were inserted proximal to gene regions (<= 2 Kb), suggesting they may have a functional influence on associated genes. In addition, microRNAs derived from MITEs may influence gene regulation which could be important for B-genome evolution (**Table S5**) (Morata et al., 2018). Furthermore, a number of potential TFBS were found in the two MITE family sequences, in particular the two BniHAT MITE families contributed 24 and 16% of LIM1 and AP3:PI motifs from the total genome, suggesting a putative role in gene regulation and stress responses (**Table S6**) (Hénaff et al., 2014). However, more functional analysis will be required to support the assumption of MITE-derived microRNA and TFBS. The abundance, genic association, and short nature of MITEs facilitates their use as simple markers in diversity and evolution studies (Sampath et al., 2015). Intact and stable inheritance of MITE can provide a source of markers for QTL and association studies (Sampath and Yang, 2014). Insertion polymorphism analysis based on MITE flanking markers provided evidence of insertion and activity in divergent B genome varieties.

## Conclusions

MITEs are an important transposon family which are present at high copy number and would be expected to impact structural and functional divergence of genes. Two hAT MITE families specific to the *B. nigra* genome were identified. Both MITE families were largely absent from the related A and C-genomes but are present at high copy numbers and have undergone relatively recent amplification in the B-genome. Though hAT family members show a random distribution throughout the genome there was a biased association with genes or gene related regions suggesting the importance of these MITEs to structural and functional evolution of the *B. nigra* genome.

## Data Availability Statement

All datasets presented in this study are included in the article/**Supplementary Material**.

## Author Contributions

SP and IP designed and contributed to the original concept of the project. SP has done the bioinformatics analysis and molecular experiments. LT helped with DNA and PCR analysis. SP and IP wrote the manuscript. SR helped with figure development. BJ, SR, SK, and T-JY helped with revision and editing of the manuscript. All authors contributed to the article and approved the submitted version.

## Funding

This work was supported by funding from the AAFC Canadian Crop Genomics Initiative and the Global Institute for Food Security.

## Conflict of Interest

The authors declare that the research was conducted in the absence of any commercial or financial relationships that could be construed as a potential conflict of interest.

## Footnotes

1
http://cruciferseq.ca/

2
http://blast.ncbi.nlm.nih.gov/Blast.cgi

3
http://www.mirbase.org/

4
http://alggen.lsi.upc.es/cgi-bin/promo_v3/promo/promoinit.cgi?dirDB=TF_8.3
