# Supplementary material for: Characterization of B-Genome Specific High Copy hAT MITE Families in Brassica nigra Genome
Source: Front Plant Sci. 2020 Jul 21;11:1104. doi: 10.3389/fpls.2020.01104 (PMC7385995; doi:10.3389/fpls.2020.01104)
Supplement: Supplementary file 1 [file DataSheet_1.docx]

**Characterization of B-genome specific high copy hAT MITE families in *Brassica* nigra genome**

**Supplementary Tables and Figures**

**Table S1.** Genome and sequencing information of the *Brassica* species used in this study

**Table S2.** Plant materials used for MITE insertion polymorphisms survey

**Table S3.** Primers and gel profile of insertion polymorphisms analysis *(in excel file )*

**Table S4.** Summary of *B. nigra* MITE super-families.

**Table S5.** Homologous micro RNAs from two MITE families

**Table S6.** Transcription factor binding sites (TFBS) from two MITE families and their distribution in different region of the *B. nigra* genome

**Table S7.** Members and position annotation of BniHAT-1 family members in the *B. nigra* genome *(in excel file )*

**Table S8.** Members and position annotation of BniHAT-2 family members in the *B. nigra* genome *(in excel file )*

**Data1 .** Sequence of BniHAT-1 and BniHAT-2 elements from *Brassica* *nigra* genome.

**Figure S1.** Sequence alignment of BniHAT-1 (A) and BniHAT-2 (B) elements from the three diploid *Brassica* genomes. Green arrows indicate the terminal inverted repeat regions.

**Figure S2.** Secondary structure of BniHAT-1 (A) and BniHAT-2 (B) elements developed using mfold (Zuker M 2003) showing possible hair-pin structures of both MITEs.

.

**Table S1. Genome and sequencing information of the *Brassica* species used in this study**

| **ID** | **Morphotype** | **Species** | **Accession (cultivar)** | **Genome** | **WGS reads** | | **Source** |
| --- | --- | --- | --- | --- | --- | --- | --- |
|  |  |  |  |  | **Amounts (Mb)** | **Coverage (x)** |  |
| Bn-1 | Black Mustard | *B. nigra* | Ni100 | BB | 2060 | 3.4 | (Perumal *et. al* 2020) |
| Bn-2 | Black Mustard | *B. nigra* | 113783 | BB | 979.8 | 1.6 |  |
| Bn-3 | Black Mustard | *B. nigra* | 113787 | BB | 979.8 | 1.6 |  |
| Bn-4 | Black Mustard | *B. nigra* | 113793 | BB | 979.1 | 1.6 |  |
| Bn-5 | Black Mustard | *B. nigra* | 113796 | BB | 981.8 | 1.6 |  |
| Bn-6 | Black Mustard | *B. nigra* | 115121 | BB | 980.8 | 1.6 |  |
| Bn-7 | Black Mustard | *B. nigra* | 115125 | BB | 980.1 | 1.6 |  |
| Br-1 | Chinese Cabbage | *B. rapa ssp. Pekinensis* | Chiifu | AA | 2321.4 | 4.4 | (Cheng *et al*. 2016) |
| Br-2 | Chinese Cabbage | *B. rapa ssp. Pekinensis* | Kenshin | AA | 1498.9 | 2.8 |  |
| Bo-1 | Cabbage | *B. oleracea ssp. Capitata* | C1176 | CC | 1541 | 2.2 | (Sampath et al. 2017) |
| Bo-2 | Cabbage | *B. oleracea ssp. Capitata* | C1220 | CC | 1606.8 | 2.3 |  |

**Table S2. Plant materials used for MITE insertion polymorphism survey**

| **ID** | **Accession (cultivar)** | **Species** |
| --- | --- | --- |
| A1 | Chiffu | *B. rapa* |
| A3 | Candle | *B. rapa* |
| A2 | Reward | *B. rapa* |
| A4 | Maleksberger | *B. rapa* |
| C1 | To1000 | *B. oleracea* |
| C3 | Gower | *B. oleracea* |
| C2 | Badger | *B. oleracea* |
| C4 | Begol | *B. oleracea* |
| B1 | Ni100 | *B. nigra* |
| B3 | 113787 | *B. nigra* |
| B2 | 113783 | *B. nigra* |
| B4 | 113793 | *B. nigra* |
| B5 | 113783 | *B. nigra* |
| B6 | 113787 | *B. nigra* |
| B7 | 113793 | *B. nigra* |
| B8 | 113796 | *B. nigra* |
| B9 | 115121 | *B. nigra* |
| B10 | 115125(C2) | *B. nigra* |
| B11 | 115137 | *B. nigra* |
| B12 | 115144 | *B. nigra* |
| B13 | 115146 | *B. nigra* |
| B14 | A1 | *B. nigra* |

**Table S4. Summary of *B. nigra* MITE super-families**

| **MITE super-family** | **#family** | **#Elements** | **Size (Mb)** |
| --- | --- | --- | --- |
| Stowaway | 97 | 8,009 | 3.5 |
| Mutator | 3 | 194 | 0.1 |
| hAT | 25 | 6,101 | 1.2 |
| Tourist | 12 | 449 | 0.2 |
| Unclassified | 33 | 3,127 | 1.3 |
|  | 170 | 17,880 | 6.3 |

**Table S5. Homologous micro-RNAs from two MITE families**

| **MITE** | **miRNA** | **MITE start** | **MITE end** | **Strand** | **E-value** | **miRBase_ID** | **Organism** |
| --- | --- | --- | --- | --- | --- | --- | --- |
| BniHAT-1 | CCGGUUGAACCGGUCCAA | 592 | 609 | - | 3.3 | gma-miR4354 | *Glycine max* |
| BniHAT-1 | UUCAUAAAAAUUAACAUAU | 164 | 182 | + | 7.2 | gma-miR1530 | *Glycine max* |
| BniHAT-1 | AUUAUAUAUAUAUAAGUUU | 550 | 568 | - | 7.2 | aly-miR4237 | *Arabidopsis lyrata* |
| BniHAT-1 | UAAAAAUUAACAUAUAAUU | 168 | 186 | + | 7.2 | gma-miR5031 | *Glycine max* |
| BniHAT-1 | UGAUUUUAUCAAUUUUA | 395 | 411 | + | 8.7 | osa-miR1438 | *Oryza sativa* |
| BniHAT-1 | UUCAUAAAAUUUGAGAU | 331 | 347 | + | 8.7 | stu-miR7984b-3p | *Solanum tuberosum* |
| BniHAT-2 | CAAAAUUAUACAUG | 208 | 221 | - | 4.9 | gra-miR8709b | *Gossypium raimondii* |
| BniHAT-2 | AUUAUACAUGUUUUUGUUU | 212 | 230 | - | 7.1 | aly-miR4237 | *Arabidopsis lyrata* |
| BniHAT-2 | UUAUACAUAUUUAAUUGUA | 433 | 451 | + | 7.1 | ath-miR5014a-3p | *Arabidopsis thaliana* |
| BniHAT-2 | AAUAUUCUUACAUAAUAUU | 234 | 252 | + | 7.1 | mtr-miR5747 | *Medicago truncatula* |
| BniHAT-2 | AUUUGCGAUUUGCUUCGUA | 41 | 59 | - | 7.1 | tae-miR9673-5p | *Triticum aestivum* |

**Table S6. Transcription factor binding sites (TFBS) from two MITE families and their distribution in different regions of the *B. nigra* genome**

| **TFBS Name** | **TFBS sequence** | **Total in Genome** | **In TE space (%)** | **In BniHAT-1 and -2 (%)** | **Function** |  |
| --- | --- | --- | --- | --- | --- | --- |
| Alfin1 | GTGTTT | 478,008 | 279,006 (58.37) | 772 (0.16) | Nucleic acid binding protein (alfin-1) | |
| AP3:PI | TTTTAGTTTAC | 1,223 | 693 (56.66) | 193 (15.78) | MADS box transcription factors | |
| C1 (long form) | TCGGATAG | 6,047 | 22 (0.36) | 2 (0.03) | General transcription factors (GTFs) | |
| DEF:GLO | CCATATTCA | 6,131 | 3,845 (62.71) | 323 (5.27) | Glo DNA-binding transcription factor activity | |
| DPBF-1 | ACACAAATAT | 4,512 | 2,206 (48.89) | 155 (3.44) | Basic Leucine Zipper (bZIP) proteins | |
| GAMYB | AACATATA | 70,038 | 31,326 (44.73) | 780 (1.11) | DNA-binding transcription factor activity | |
| GT-1 | ATTAACAT | 38,445 | 17,171 (44.66) | 347 (0.9) | Trihelix transcription factor GT-1 | |
| GT-1b | ATTTGTAAAAA | 3,021 | 1,849 (61.2) | 238 (7.88) | Trihelix transcription factors | |
| HSF1 | GTGTT | 1,185,551 | 671,389 (56.63) | 1,278 (0.11) | Heat shock factor 1 (HSF1) | |
| LIM1 | CCAACCAGTC | 982 | 771 (78.51) | 231 (23.52) | Cysteine-rich zinc-binding domain | |
| MNB1a | ATACTTTTTA | 5,446 | 2,658 (48.81) | 33 (0.61) | Dof zinc finger protein MNB1A | |
| MYB2 | TGTT | 6,698,523 | 3,502,398 (52.29) | 5,629 (0.08) | Abiotic stress responses | |
| PF1 | TATTACTA | 30,949 | 14,704 (47.51) | 236 (0.76) | Homeodomain zinc finger protein | |
| PHR1 | ACCATATTC | 7,582 | 6,278 (82.8) | 270 (3.56) | Pi-starvation response | |
| SBF-1 | CAATTTTAAAA | 3,812 | 1,932 (50.68) | 324 (8.5) | SBF1 (SET Binding Factor 1) | |
| SPF1 | TAATATT | 300,919 | 151,715 (50.42) | 1,240 (0.41) | Ion-transporting P-type ATPase | |
| SQUA | ACCATTTA | 25,386 | 12,895 (50.8) | 4 (0.02) | DNA-binding transcription factor | |
| TRM1 | TATTTTCT | 99,350 | 46,890 (47.2) | 300 (0.3) | Zn(II)2Cys6-type transcription factor Trm1 | |

**Data 1.** Sequence of BniHAT-1 and BniHAT-2 elements from *Brassica* *nigra* genome.

>BniHAT1

CAGTGTTTTGAAAACCGGACCGGACCAGCCGGTCGAACCGGTTCGACCGTGACTCGCTGAAGAAGCCGAGTCCGGTTCGATTTAAAACCCGGATTTGTAAAAAACCGGTAAAACCGGCTGAAAACCGGTAAAACCGGTGACCCGGTTCAATTTTAAAACCCGGTTCATAAAAATTAACATATAATCAAGTGTTTTCAATTATTACTAGCTAAAACTTAATATTTTATAATTAGTGTTCAACTATCACCATTTAATTAATTTTTTATTTTCTTAAGAAACAATGCGAAAAATATATTTTTGATTTTCATATACTATCTTTATTTTACCATATTCATAAAATTTGAGATTTAGCTGTAAAAATAAATAAATATGATAATTTAATTATAAATCATATGATTTTATCAATTTTAGTTTACATAGTATTCACTATTCAGTTTTATTTTACTATATATACAGTTAGATTTATGTTTATTTTTTTATATTTAGTTAATTACAGAAAAGAATACAGAATGTATTATATGTAGAAGTAAAATTCATATAATATATATATAATTATATATATATATATATATAAGTTTCATAGATAGAAACCCGGTTCGACCGGTTGAACCGGTCCAACCAGTCAACCAGCAGGTACACCGAGTCGGTATCCGGTCCGGTTTTCAAAACATTG

>BniHAT2

TAGGGGTGGGCACGGATCGGATAGTACCATATTTTTTGGTATTTGCGATTTGCTTCGTAAGTTACGAATATCTGATTTTCCGATTTGTTTTGCTTCGGAGAAATACGTATATTCGGTTTTCCGGATATTCGGAATTTTTTTAATTATTTGCGAATATTTACGGATATTTACGAATATCTCATACACCATGTTTTATACACGTAAATCTAACAAAATTATACATGTTTTGTTTTGTTTTTTAAATATTCTTGCATAATATTAAACAAAAAATGAAAATTAGTGAAATTATATGTTCTATACTTTTTATAATAAAATAATCTTTTTTGATAAAACACATAATTTTAGAAAATTATAGTTTTTTGAAAGATTTTATATTCTTTTCTTAATTTAATACATTATATATTTTTATAAATAACACAAATATATGTTACAGTAAAAATTTATACATATTTAATTGTAGATATTCATTTATTTGTATAAAAACCGGAGCGGATCGGATATTCGCCTTTAAAATTTTTAGTATTTATGATTTGCTTCGTTTTTAACGGATATTGAATTTTAATATTTACTTTGCTTCGAAGACTTACGGAATATTCGGATTTTTCGGATCAAATCGAAACGAATAGCGAATCGAATCAAATTTAACGGATAAAATGCCCACCCCTA


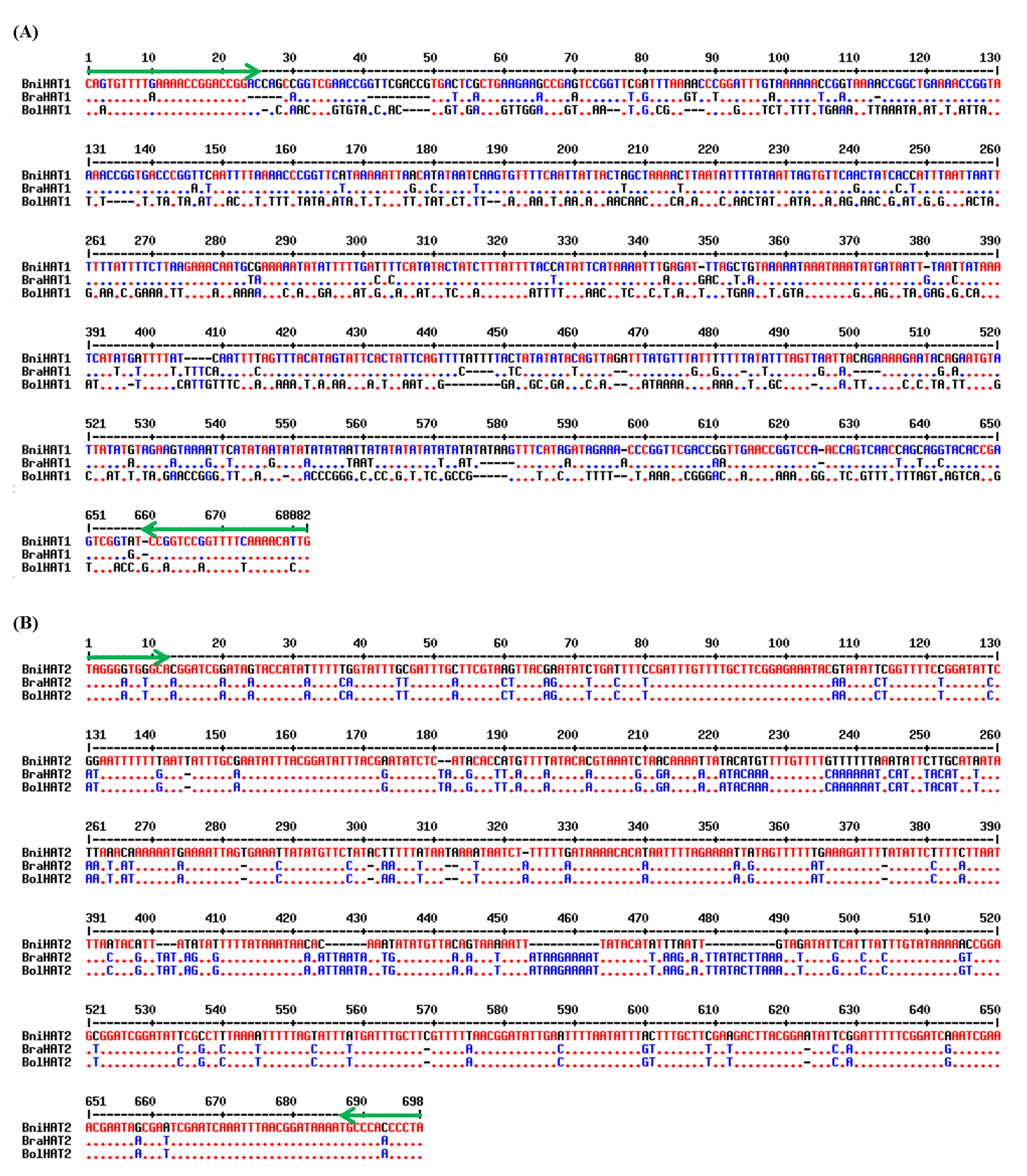

**Figure S1.** Sequence alignment of BniHAT-1 (A) and BniHAT-2 (B) elements from three diploid *Brassica* genomes. Green arrows indicate the terminal inverted repeat regions.


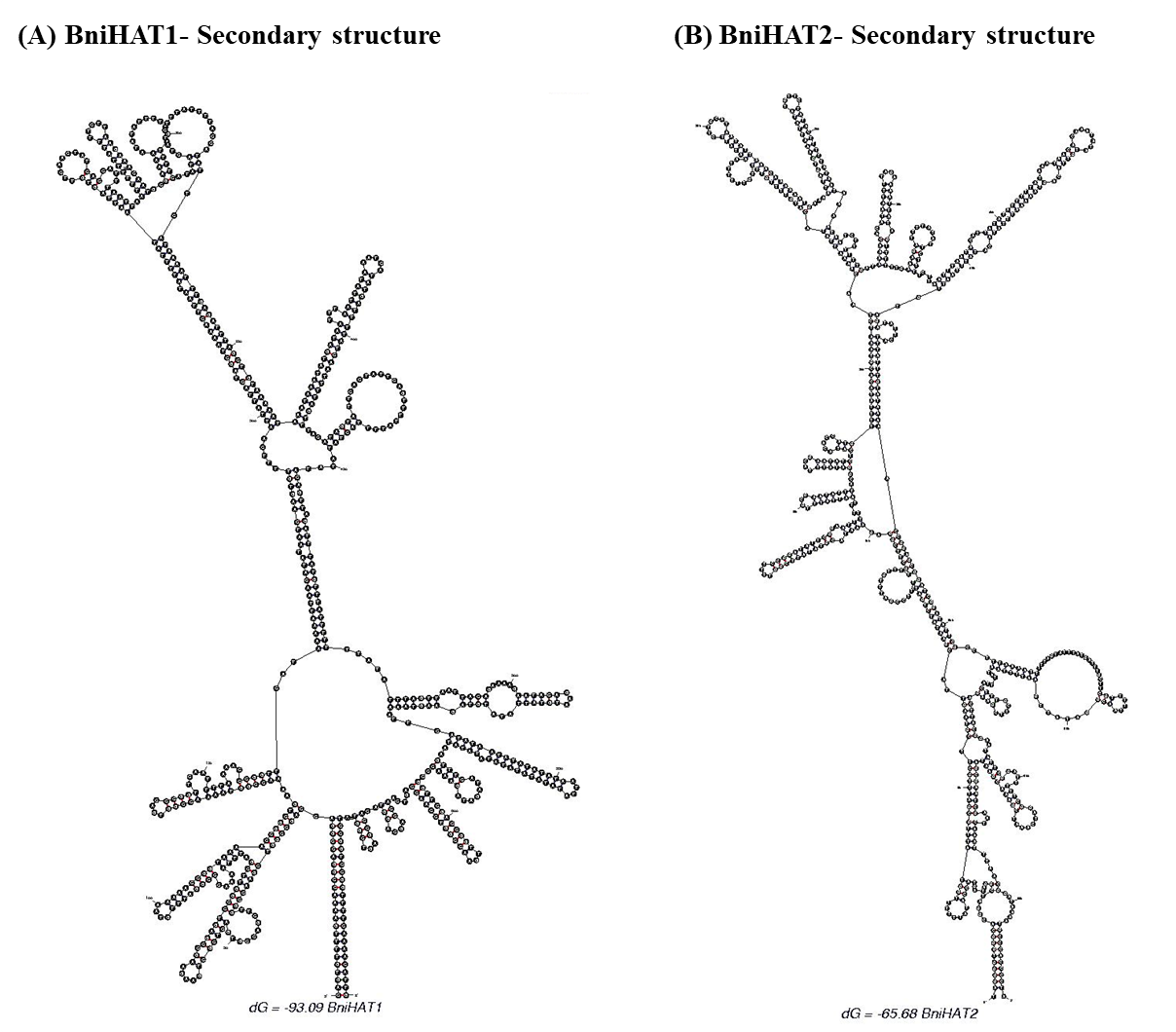


**Figure S2.** Secondary structure of BniHAT-1 (A) and BniHAT-2 (B) elements developed using mfold (Zuker M 2003) showing possible hair-pin structures of both MITEs.
